# In-situ formation mechanism of endogenous fluorescent carbon dots during the roasting process of small yellow croaker *(Larimichthys polyactis*)

**DOI:** 10.1016/j.fochx.2025.102187

**Published:** 2025-01-17

**Authors:** Yutong Qu, Shuyi Zhao, Jilong Ni, Long Jiao, Xiaoye Zhang, Soottawat Benjakul, Zhiyu Liu, Xiang Chen, Bin Zhang

**Affiliations:** a Key Laboratory of Health Risk Factors for Seafood of Zhejiang Province, College of Food Science and Pharmacy, Zhejiang Ocean University, Zhoushan 316022, China; b Pisa Marine Graduate School, Zhejiang Ocean University, Zhoushan 316022, China; c School of Naval Architecture and Maritime, Zhejiang Ocean University, Zhoushan 316022, China; d International Center of Excellence in Seafood Science and Innovation, Faculty of Agro-Industry, Prince of Songkla University, Songkhla 90112, Thailand; e Key Laboratory of Cultivation and High-value Utilization of Marine Organisms in Fujian Province, Fisheries Research Institute of Fujian, Xiamen 361013, China; f Zhoushan Institute for Food and Drug Control, Zhoushan 316021, China.

**Keywords:** Fluorescent carbon dot, Small yellow croaker, Roasting, Formation mechanism, In-situ analysis technique

## Abstract

The endogenous fluorescent carbon dots (FCDs) existed in roasted foods, leading to extensive attention due to their physicochemical properties and potential biotoxicity. Therefore, the in-situ formation mechanism and physicochemical properties of endogenous FCDs in roasted small yellow croaker (*Larimichthys polyactis*) were investigated. The fluorescence spectrum of FCDs underwent a blue-shift with an increase in the roasting temperature. Scanning electron microscopy/energy dispersive spectrometry (SEM/EDS) analysis revealed that FCDs primarily consisted of C and O, exhibiting significant variations across different roasting temperatures (180 °C, 200 °C, and 220 °C). The in-site variable temperature-Fourier transform infrared (VT-FTIR) spectrum demonstrated notable changes as the temperature increased from 26 °C to 220 °C. This was due to the thermal aggregation of biological macromolecules such as proteins and fats, leading to thermal decomposition and disintegration, resulting in the formation of FCDs (∼5 nm). These results provided a theoretical basis for controlling the formation of FCDs in aquatic products during heat processing.

## Highlights

• Endogenous fluorescent carbon dots (FCDs) were extracted from roasted L. *polyactis*.
• The elemental composition of FCDs was influenced by varying roasting temperatures.
• The in-situ FCDs involved thermal aggregation, decomposition, and disintegration.

## 1 Introduction

Carbon dots (CDs) are typically carbon-based nanoparticles smaller than 10 nm and mainly consist of carbon, nitrogen, oxygen, and hydrogen. And the endogenous fluorescent CDs (FCDs) are widely present in food items such as bread, coffee, animal meat, and aquatic products (Anpalagan et al., 2020; Cui et al., 2022; Jeong et al., 2023; Li et al., 2023). Because of their optimal nanosize, exceptional photoluminescence properties, and high photosensitivity, endogenous FCDs have garnered significant attention in the fields of biofluorescence imaging (Kang et al., 2015; Yin et al., 2024), photodynamic inactivation (Dong et al., 2021), and photocatalytic degradation (Venugopalan et al., 2023). However, endogenous FCDs formed during the high-temperature processing of food may potentially exert detrimental effects on biological systems (Nel et al., 2015). Therefore, investigating the physicochemical properties of endogenous FCDs and their formation processes in food substrates is of significant importance.

Frying and roasting are the predominant thermal processing methods for economically significant aquatic plants and animals, including fish, shrimp, crabs, shellfish, and algae (Aubourg, 2018). Particularly during high-temperature processing, a significant number of endogenous FCDs may be formed (Li et al., 2019). Song et al. reported endogenous FCDs with different fluorescence properties and particle sizes in roasted chicken at different temperatures (Song et al., 2018). The results showed that endogenous FCDs could enter the cytoplasm of HepG2 cells and subsequently traverse the blood-brain barrier of BALB/c mice. Notably, food-borne FCDs formed at elevated temperatures exhibited more pronounced cytotoxic effects than those formed at lower temperatures. Huang et al. extracted FCDs from toasted shrimp and evaluated their biological characteristics (Huang et al., 2024). The results showed that FCDs' size decreased as the temperature and time of the C=O/C-H bond breakage increased. The contents of Na^+^, K^+^, and Mg^2+^ exhibited significant simultaneous increases. Furthermore, endogenous FCDs may degrade the biological characteristics of hemoglobin. Therefore, it is imperative to investigate the structural characteristics and potential biosafety of endogenous FCDs (Wang et al., 2020). This will establish a crucial foundation for investigating the regulation of endogenous FCDs formation in foods during thermal processing.

Although the preparation and structural properties of endogenous FCDs in food have been extensively studied, the in-situ formation mechanism of endogenous FCDs in aquatic products during thermal processing has not yet been elucidated. Herein, the present study investigated the structure and spectral characteristics of endogenous FCDs prepared by the little yellow croaker (*L. polyactis*) at different roasting temperatures (180, 200, and 220 °C). The formation process and rule of endogenous FCDs at the different roasting temperatures were further investigated through scanning electron microscope/energy dispersive spectroscopy (SEM/EDS) and transmission electron microscope (TEM) analysis, as well as in-situ variable temperature-Fourier transform infrared (VT-FTIR) Spectrometer. Based on this, a mechanism for the in-situ formation of endogenous FCDs is proposed. This study provides a significant theoretical foundation and is a valuable reference for understanding the in-situ formation mechanism and control strategies of endogenous FCDs in thermally processed aquatic products. Furthermore, this study also establishes a foundation for the controlled preparation of food-derived bioluminescence imaging materials.

## 2 Materials and methods

### 2.1 Materials and chemical reagents

***L. polyactis*was purchased from a local market in Zhoushan, China. Dialysis bags with a molecular weight cut-off of 500 Da were purchased from JielePu Co., Ltd. (Zhoushan, China). All other chemicals and solvents used in this study were of analytical grade. Ultrapure deionized water from a Milli-Q ultrapure system (UPR-I-5TNZP, Sichuan UPR, China) was used for all the analysis, separation, and purification steps.**

### 2.2 Extraction of FCDs from roasted L. *polyactis*

**Fig. 1:**
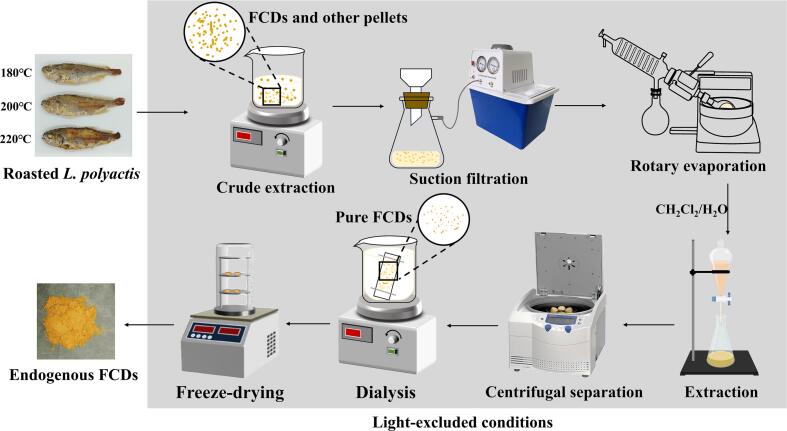
The preparation process of endogenous FCDs.

The preparation process for the endogenous FCDs was shown in Fig. 1. **The internal organs and scales of raw** L. ***polyactis*were removed and then washed three times with ultrapure deionized water. After eli**min**ating surface moisture, *L. polyactis*was roasted in an electric heating oven (NN-GF351H, Shanghai Panasonic, China) at different roasting temperatures (180, 200, and 220** °C**) for 40 min. The selection of roasting temperatures was determined based on considerations of the traditional roasting process and the resulting flavor post-roasting. Furthermore, according to previous studies, roasting temperatures within the range of 180** **°C to 220** **°C frequently led to the formation of endogenous FCDs (****Cui et al.,** 2022**;** **De & Karak,** 2013**;** **Huang et al.,** 2024**). FCDs were extracted from roasted** L. ***polyactis*** based on a previously reported extraction method with some modifications (Bi et al., 2018; Ke, 2019). The entire process of FCDs extraction from roasted L. *polyactis* was performed under light-free conditions. Briefly, the roasted L. *polyactis* was added to ethanol (w: v = 1: 10), and then stirred with a magnetic mixer for 24 h. Next, the collected initial extract was evaporated by rotation to remove ethanol. The crude extract was subsequently subjected to extraction with a mixture of equal volumes of methylene chloride and ultrapure deionized water, followed by the collection of the aqueous layer. The supernatant was obtained by centrifuging the aqueous layer at 10619 ×g for 10 min. The supernatant was filtered using a 0.22 μm filter membrane, followed by dialysis for 48 h. Finally, the resulting dialysate was collected and freeze-dried for 48 h to obtain yellow solid endogenous FCDs.

### 2.3 Ultraviolet-visible (UV–vis) absorption and fluorescence spectroscopy analysis

Working solutions were prepared by dissolving endogenous FCDs in ultrapure deionized water. UV–vis absorption (scan: 200–900 nm, step increment: 2 nm, and band pass: 5 nm) and fluorescence spectra (excitation wavelength: 300 nm, emission range: 320–900 nm, and excitation band pass: 5 nm) were recorded using an absorption and fluorescence spectrometer (Duetta, Horiba, Japan) at room temperature. The absorption and fluorescence spectra were generated using Origin9.0 software.

### 2.4 Fluorescence microscopic imaging analysis

The solid in situ and purified endogenous FCDs were pressed on a slide and cover slide and then observed under a fluorescent inverted microscope (AxioVert5, ZEISS, Germany). The fluorescence detection range was 420–490 nm using a purple laser source (λ_ex_ = 365–395 nm) and a 395 nm filter.

### 2.5 In-site VT-FTIR spectroscopy analysis

The variable temperature properties of the samples were examined using a VT-FTIR spectrometer (Thermo IS 50, Thermo Fisher, USA). The processed fish samples were placed in the ATR unit, where they were gradually heated to 26, 60, 100, 150, 180, 200, and 220 °C at 30 mL/min of airflow. Each temperature gradient was maintained for 30 s during the collection of the corresponding VT-FTIR spectra. The resolution was 4 cm^−1^, with 32 scans spanning the wavenumber range of 650–4000 cm^−1^.

### 2.6 SEM/EDS and TEM characterization

Endogenous FCDs were bonded directly to the conductive adhesive, and gold was sprayed for 45 s at 10 mA using a sputtering coater (SC7620, Quorum, UK). The morphology of the samples was photographed using a scanning electron microscope (Sigma 300, ZEISS, Germany), and energy spectrum mapping was performed. The acceleration voltage during shooting was 3 kV, while that during spectral mapping was 15 kV. A SE2 secondary electronic detector was used. TEM analysis of endogenous FCDs was performed using a transmission electron microscope (JEM-2100F, JEOL, Japan) at a voltage of 200 kV.

### 2.7 Stability evaluation

#### 2.7.1 The assessment of pH value

The endogenous FCDs (0.5 mg) were dissolved in 5 mL of 0.1 mol/L HCl, followed by pH adjustment to 12 using a 0.1 mol/L NaOH solution. The transmittance of the sample solution at different pH values was measured at 600 nm using an ultraviolet-visible spectrophotometer (T600, Purkinje, China).

#### 2.7.2 The assessment of fluorescence stability

Endogenous FCDs (1.0 mg) were dispersed in 10 mL of ultrapure deionized water via ultrasonic treatment (40 kHz, 10 min) and subsequently placed indoors under natural light conditions. The fluorescence spectra of the solutions were measured on days 0, 1, 3, 5, and 7 using absorption and fluorescence spectrometers (Duetta, Horiba, Japan). The spectra were normalized using Origin9.0 software.

### 2.8 Data analysis

The results obtained from the three replicates of each determination were subjected to statistical analyses using SPSS 22 for Windows (SPSS Inc., Chicago, IL, USA). Duncan's test was conducted to determine the significant differences among the various treatments, and a *P*-value of less than 0.05 was adopted to indicate that the means differed significantly.

## 3 Results and discussion

### 3.1 Optical property analysis

**Fig. 2:**
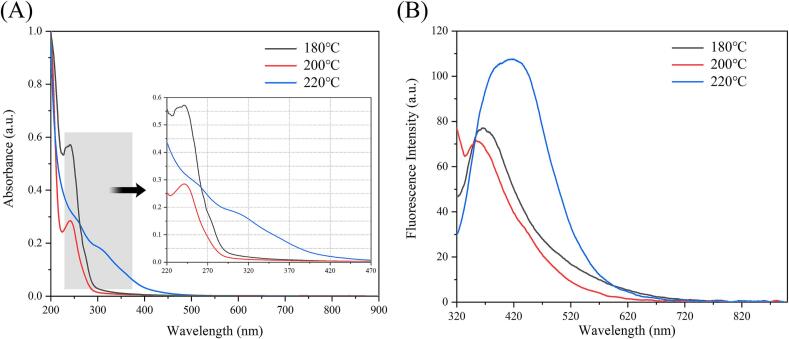
(A) UV–vis absorption and (B) fluorescence spectrums of CDs.

UV–vis absorption and fluorescence spectroscopy were employed to investigate the optical properties of the endogenous FCDs. The UV–vis absorption spectrum (Fig. 2A) demonstrated that the absorption peak underwent a significant redshift and reduction in intensity as the roasting temperature increased. The endogenous FCDs exhibited significant absorption at the short wavelength (∼240 nm) at the roasting temperatures of 180 °C and 200 °C. However, the appearance of a new weak absorption peak (∼300 nm) was observed at the roasting temperature of 220 °C, which could be attributed to the π-π* electron transition in the C=C and bond and the n-π* electron transition in the C=O bond (De & Karak, 2013). This also indicated that the endogenous FCDs had a sp^2^ carbon structure and the presence of C—O or C—N functional groups (Xiaoming & Yan, 2014; Zhu et al., 2019). With increased roasting temperature, the carbon content within the endogenous FCDs might progressively increase, whereas the oxygen content diminishes. Consequently, this led to the breakage of the C—N and/or C—O bonds, weakening their shortwave absorption peaks (∼240 nm) (Huang et al., 2024).

At an excitation wavelength of 300 nm, the endogenous FCDs exhibited distinct fluorescence characteristics. As depicted in Fig. 2B, with increasing roasting temperature, the maximum emission wavelength of the FCDs shifted from 350 to 420 nm. This phenomenon can be attributed to the augmentation of sp^2^ conjugates within the FCDs formed at elevated temperatures, leading to alterations in the π-electron energy level. Naturally, this redshift was also influenced by particle size and solvent environment (Shuang et al., 2019; Sk et al., 2012). Additionally, the fluorescence intensity significantly increased as the roasting temperature increased owing to the concurrent augmentation in the number of fluorophores within the FCDs matrix (Saxena & Sarkar, 2014; Yan et al., 2019).

**Fig. 3:**
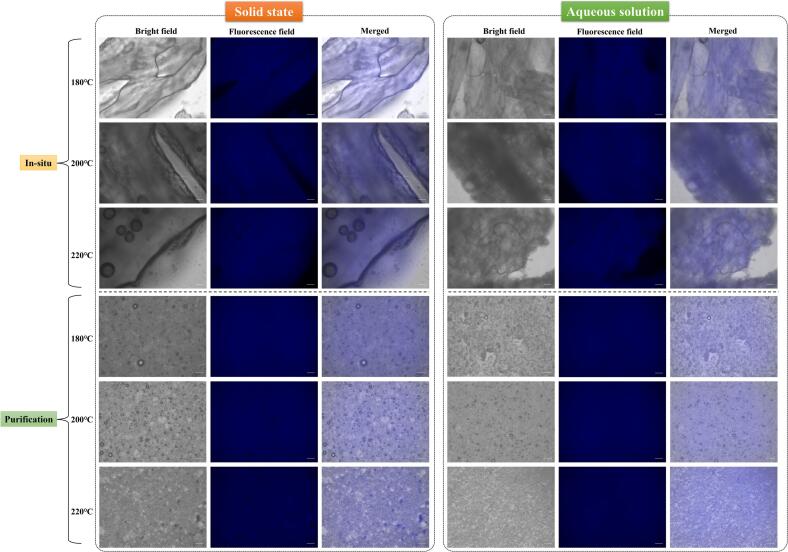
Fluorescence microscopic images (magnification 200×) of (A) in-situ fish samples and (B) purified endogenous FCDs.

In-situ samples and purified endogenous FCDs exhibited remarkable fluorescence properties in both solid and solution states (Fig. 3), indicating the ability of L. *polyactis* to generate endogenous FCDs at low roasting temperatures (180 °C). Spherical aggregate particles were clearly observed in the in-situ samples at high roasting temperatures. Furthermore, upon increasing the roasting temperature, a gradual decrease in the particle size of the endogenous FCDs was observed, along with a uniform fluorescence signal.

### 3.2 In-situ formation analysis of the endogenous FCDs

**Fig. 4:**
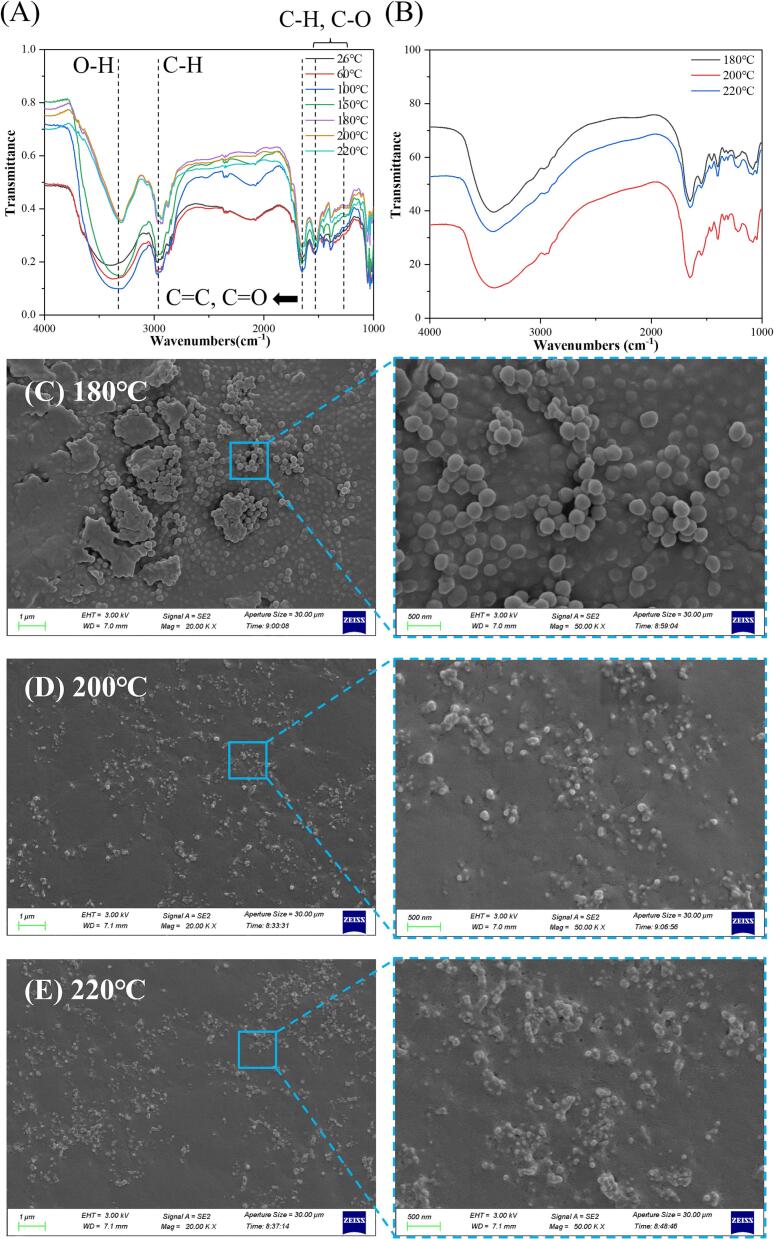
(A) The in-site VT-FTIR spectrum of in-situ fish samples, (B) the VT-FTIR spectrum of purified endogenous FCDs, and (C - E) the morphology of in-situ fish samples at different roasting temperatures.

The in-situ VT-FTIR spectra of the fish samples were subsequently measured and compared with those obtained from purified endogenous FCDs. The VT-FTIR spectrum in Fig. 4A revealed a significant increase in the infrared transmittance of the in-situ fish samples with increasing roasting temperatures. This observation suggested that the functional groups or chemical structures inside the fish undergo changes during the formation of the original FCDs, resulting in a weakened absorption capacity for infrared light. The results showed that the presence of hydroxyl groups was reflected by the broad peak seen at about 3384 cm^−1^, which might be due to the stretching vibrations of O—H. The presence of the C—H stretching vibration of saturated carbon was signified by the peak observed at 2965 cm^−1^; it might also have stretching vibrations of -CH_3_ and -CH_2_ (Song et al., 2019). The peak at ∼2000 cm^−1^ could diminish gradually with the increase in temperature and disappear entirely at 180 °C. This temperature increase might lead to the degradation of biomolecules containing unstable double and triple bonds. The bending vibrations of the carbonyl groups or C=C bonds were observed at 1650 cm^−1^, while the absorption peaks at 1545 cm^−1^ indicated the formation of C—O structures (Wang et al., 2019). Additionally, when the temperature exceeded 150 °C, the characteristic bending vibrations of C—H bonds emerged at 1300–1500 cm^−1^, indicating the formation of a single-bond molecular structure (including C—H and C—O) due to unsaturated bond rupture at high temperatures. The VT-FTIR spectra of the purified endogenous FCDs (Fig. 4B) also exhibited identical positions for the characteristic peaks observed in situ, particularly the representative bending vibrations of the newly generated single-bond structures (C—H and C—O) (Alam et al., 2015). Consequently, a stable structure of the endogenous FCDs could be formed in L. ***polyactis*** at 220 °C.

**Fig. 5:**
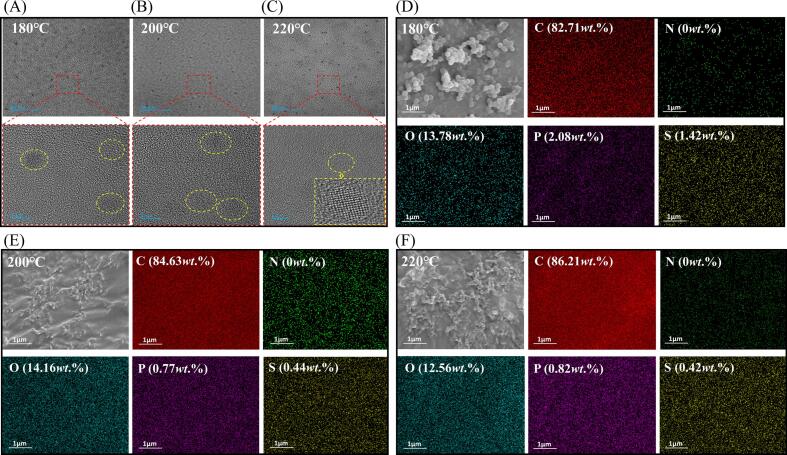
(A - C) TEM images and (D - F) elemental EDS maps of purified endogenous FCDs.

Fish samples were analyzed using SEM to further visualize the in-situ formation process of endogenous FCDs at different roasting temperatures. After being roasted at 180 °C (Fig. 4C), the fish samples exhibited a coarse texture and prominent aggregation of large spherical particles. With increased roasting temperature (Fig. 4D), these larger particles gradually disintegrated, forming smaller, irregular particles. At a roasting temperature of 220 °C (Fig. 4E), these small particles underwent further pyrolysis to generate minuscule particles, including FCDs. Purified endogenous FCDs prepared at different roasting temperatures were subsequently analyzed using TEM. Compared with Fig. 5A - 5C, it could be observed that the endogenous FCDs formed at a lower roasting temperature (180 °C) exhibited larger sizes and lacked distinct lattice characteristics. However, as the roasting temperature increased to 220 °C, there was a noticeable enhancement in the contrast of the endogenous FCDs and a significant reduction in particle size (∼5 nm), accompanied by the emergence of an ordered lattice structure (as depicted in Fig. 5C upon local zooming) (Kundu et al., 2018; Shoujun Zhu et al., 2012).

To further explore the structural differences, SEM/EDS analyses of the endogenous FCDs formed at different roasting temperatures were conducted (Fig. 5D -5F). The EDS maps revealed that the endogenous FCDs primarily consisted of substantial amounts of carbon, oxygen, and trace amounts of phosphorus and sulfur. As the roasting temperature increased, the carbon content significantly increased. In contrast, the oxygen content remained relatively stable, indicating the structural stability of the FCDs. Furthermore, the phosphorus and sulfur levels decreased with increasing temperature, suggesting the rapid decomposition of biological macromolecules such as proteins and phospholipids at high roasting temperatures. Notably, nitrogen was not detected at any of the three roasting temperatures, indicating severe thermal degradation of proteins at 180 °C. The elemental compositions of the FCDs formed at different roasting temperatures exhibit distinct variations, which account for their different spectral characteristics.

### 3.3 Stability evaluation

**Fig. 6:**
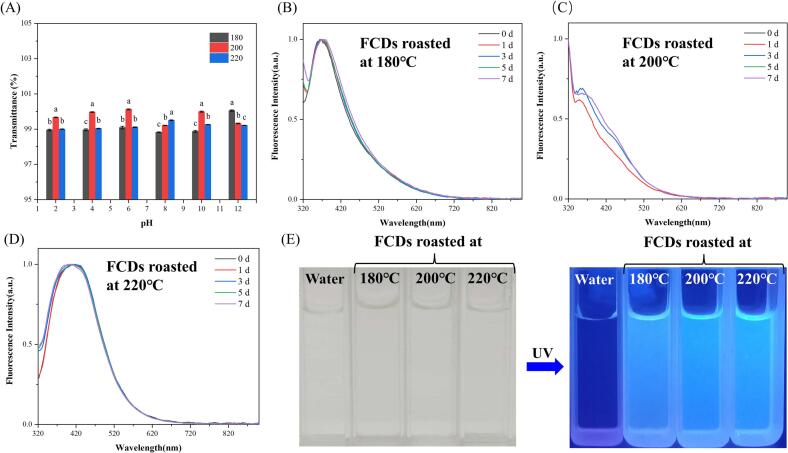
(A) The dispersion stability and (B - D) fluorescence evaluation of purified endogenous FCDs. (E) Fluorescent images of FCDs after 7 days under indoor natural light.

The stability of endogenous FCDs is a crucial prerequisite for their biological application and one of the key factors influencing their biological toxicity (Omran et al., 2021). Therefore, an assessment was conducted to evaluate the pH and fluorescence stabilities of the endogenous FCDs in an aqueous solution. The results were presented in Fig. 6. As shown in Fig. 6A, endogenous FCDs prepared at different roasting temperatures exhibited excellent fluorescence stability over the entire pH range. The normalized fluorescence spectra in Fig. 6B - 6D revealed that the fluorescence of the endogenous FCDs under indoor natural light for 7 days remained essentially unchanged, indicating their exceptional fluorescence stability (Fig. 6E). Consequently, its outstanding stability in aqueous solutions served as a crucial foundation for its application as a biofluorescent imaging material. However, their exceptional stability might enhance their potential toxic effects in vivo.

### 3.4 Formation mechanism of endogenous fluorescent CDs in L. *polyactis*

**Fig. 7:**
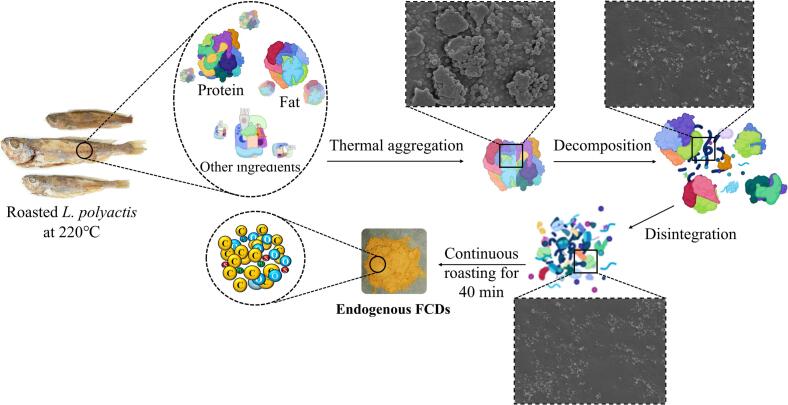
The in-situ formation mechanism of the endogenous FCDs in L. ***polyactis***.

Based on the aforementioned research on endogenous FCDs, we proposed a potential mechanism for the formation of endogenous fluorescent FCDs during the roasting of L. ***polyactis***, as illustrated in Fig. 7. During the roasting stage, heat denatured the protein and fat in L. ***polyactis***, causing them to aggregate into larger particles, which was accompanied by a decrease in protein and fat content (Rose et al., 2015). As the roasting temperature reached 200 °C, these large protein and fat particles underwent further thermal decomposition reactions, gradually forming micron-sized particles. When the roasting temperature rose to 220 °C, these particles accelerated their disintegration process, forming nanoscale particles. After continuous roasting at 220 °C for 40 min, these nanoscale particles ultimately underwent accelerated thermal disintegration to form monodisperse endogenous FCDs predominantly composed of carbon and oxygen elements.

## 4 Conclusions

In conclusion, this study investigated the temperature effect on the formation endogenous FCDs with varying the structural and fluorescence properties in L. *polyactis* at different roasting temperatures. It was confirmed that different roasting temperatures significantly affected the elemental composition and fluorescence characteristics of endogenous FCDs. In addition, it was postulated that the formation of endogenous FCDs occurs through thermal aggregation, thermal decomposition, and thermal disintegration, as indicated by in situ measurements. In a subsequent study, we will further investigate the variance in biotoxicity among endogenous FCDs with distinct structural and elemental compositions. The objective is to determine optimal thermal processing conditions for aquatic products that can effectively impede the formation of endogenous FCDs during the roasting procedure. In addition, this study also provides potential food-derived functional materials for sensing and biofluorescence imaging.

## CRediT authorship contribution statement

**Yutong Qu:** Writing – original draft, Validation, Data curation. **Shuyi Zhao:** Validation, Data curation. **Jilong Ni:** Validation, Data curation. **Long Jiao:** Writing – review & editing, Project administration, Funding acquisition. **Xiaoye Zhang:** Writing – review & editing. **Soottawat Benjakul:** Validation, Supervision. **Zhiyu Liu:** Validation, Supervision. **Xiang Chen:** Project administration, Funding acquisition. **Bin Zhang:** Validation, Supervision, Project administration, Funding acquisition.

## Declaration of competing interest

The authors declare that they have no known competing financial interests or personal relationships that could have appeared to influence the work reported in this paper.

## Data Availability

Data will be made available on request.
